# Genetics of Obesity and Type 2 Diabetes in African Americans

**DOI:** 10.1155/2013/396416

**Published:** 2013-03-19

**Authors:** Shana McCormack, Struan F. A. Grant

**Affiliations:** ^1^Division of Endocrinology, The Children's Hospital of Philadelphia, Philadelphia, PA 19104, USA; ^2^Division of Human Genetics, The Children's Hospital of Philadelphia Research Institute, Philadelphia, PA 19104, USA; ^3^Department of Pediatrics, University of Pennsylvania School of Medicine, Philadelphia, PA 19104, USA; ^4^Center for Applied Genomics, The Children's Hospital of Philadelphia Research Institute, Philadelphia, PA 19104, USA

## Abstract

Obesity and type 2 diabetes are highly prevalent and lead to significant morbidity and mortality. In the United States, the impact of these conditions may be worse on historically underserved minorities, particularly African Americans. Genetic ancestry and differences in physiology are unlikely to be the sole or primary determinants of these disparities. In addition, research in this area has the ethically problematic possibility of conflating race with biology. Despite these important considerations and the challenges of conducting this work, population-based approaches for investigating the etiology of obesity and T2D may yield useful information about the pathophysiology of disease, and have implications that extend to all affected individuals. The purpose of this paper is to describe what is understood about the genetic variation that underlies obesity and T2D in African Americans and other individuals of more recent African descent and to highlight several examples that illustrate how ensuring adequate minority representation in genetic research improves its quality. For a variety of reasons a number of unique insights have been possible as a result of these efforts.

## 1. Introduction

In this paper, we will use the previously formulated definition of race by the Endocrinology Society's 2012 consensus statement on health disparities in endocrine disorders as a “complex multidimensional construct reflecting the confluence of biological factors and geographical origins, culture, economic, political, and legal factors, as well as racism” [1]. As described in more detail below, in genetic research, individuals are often grouped on the basis of markers of ancestry and admixture; these delineations may help to address the important problem of population stratification in association studies. Results are frequently reported with respect to commonly used racial designations, a practice that is intended to make findings understandable and generalizable, but that may fail to appropriately emphasize the distinction between race and biology.

A full discussion of the methodological and ethical issues involved in using racial categories in genetic research is beyond the scope of this paper; the reader is referred to a number of excellent papers on this topic [2]. A multidisciplinary working group previously convened to address this issue emphasized that each individual has characteristics that could form the basis for his or her membership in any number of populations [3]. This group recommended that, in research publications, methodology should be clear and justification provided for classifying individuals with respect to race, and the sociocultural and ethical implications of the work should be addressed. In this paper, we will summarize these parameters in the primary data to the best extent possible; as an example, a recent study carefully surveyed the published literature on the role of PPAR*γ* polymorphisms in various racial groups in T2D and concluded that there is room for improvement in reporting and supporting the methods used to designate racial groups [4]. 

## 2. Race and Ethnicity in Genetic Studies

Methodological innovations have been critical for leveraging multiethnic studies to produce novel findings. These will be summarized here; interested readers are referred to additional references [5–10].

As noted in the previously cited reviews, most large-scale genomic association studies have been conducted historically in individuals of European descent, and, more recently, in Asians as well. There is an increasing recognition of the value of extending this work into individuals of African, as well as Hispanic or Latin American, descent. The unique challenges of performing research in groups with significant genetic diversity and a complex population history [11] may be better viewed as opportunities for new insights. A brief introduction to these issues is warranted, as they affect the interpretation of the genetic studies that have been performed to date.

First, the degree of linkage disequilibrium (LD) may be less in individuals of recent African descent because of the longer time to common ancestors [23]. Linkage disequilibrium is the nonrandom association of alleles at adjacent loci; put another way, LD refers to the size of the “chunks” of genetic material that tends to be inherited together. Genome-wide association studies (GWAS) take advantage of LD and seek to find genetic variants, most often single-nucleotide polymorphisms (SNPs), that associate with disease. Rarely, the SNP itself is considered disease causing, but, more often, it is merely a genetic proximity for the pathologic variant, and the challenge becomes pinpointing the causal allele. In individuals with a lesser degree of LD, like many of African descent, fewer SNPs may associate with disease-causing alleles, potentially reducing the yield of genome-wide association studies (GWAS). Viewed from an alternate perspective, the SNPs that do associate with disease are more likely to localize much closer to the pathologic regions. In at least two cases discussed in this paper, namely, *FTO* with obesity and *TCF7L2* with diabetes, studies in populations of African descent have facilitated the identification of likely causal variants. 

Accounting for the extent of LD, allelic heterogeneity (that may arise in different groups (i.e., when a number of different mutations at the same locus may cause the same disease) improves the yield of meta-analyses [24]. For example, in a study of south-east Asians, application of a region-based strategy mindful of these considerations yielded a novel associated loci with T2D in a region harboring *STK39,* as well as another including *GNG2* and *NID2*, that were subsequently replicated in a separate cohort [25].

Genetic heterogeneity, as well as the lack of accurate reference data, may also make a process called genotype imputation more difficult in individuals of more recent African descent. In GWAS, not all genotypes are measured directly; those that are missing can still be studied in relation to disease if they are imputed, or inferred, based on knowledge about how they relate to those genotypes whose identities have been determined [26]. This process requires the use of reference panels, groups of individuals in whom genetic information is densely characterized. The advent of specific panels in the HapMap consortium for individuals of recent African descent can improve the accuracy of imputation strategies, and the quality of GWAS and meta-analyses [27], with data from the 1000 Genomes project becoming increasingly invaluable as well in this regard [28]; indeed, these strategies are also considered useful in Latinos [29]. In addition, the phenomenon of phenotypic heterogeneity may be particularly relevant in diverse populations. As discussed in subsequent sections, the same BMI may reflect a different degree of adiposity in distinct racial groups [30]. The fact that the BMI threshold used between studies to define overweight or obesity may also vary further complicates this problem. 

Another consideration in GWAS is how to best account for a challenge referred to as “population stratification” [31]. Within a population, there may be groups who differ in the prevalence of a disease of interest. The groups may also differ in other ways, for example, in allele frequencies for genes entirely unrelated to the condition of interest, including some related to their ancestry. When cases and controls are compared, population stratification may led to the detection of spurious associations. This issue is particularly salient for studies of obesity and type 2 diabetes, whose prevalence differs between racial groups. In formulating solutions to account for population stratification, issues including diverse and overlapping ancestral origins, assumptions about random mating, migration, and geographical isolation result in additional complexity. A commonly used approach employs principal components analysis to model differences between groups and account for these differences [32]. More recent mixing between populations of divergent genetic ancestry is referred to as genetic admixture, and its relevance is the subject of investigation as well. For example, in the Atherosclerosis Risk in Communities (ARIC) study, adjustment for ancestral genetic markers in self-identified black individuals allowed the identification of a locus on 2p23.3 where there was dose response between the number of protective alleles and BMI (0.92 kg/m^2^) [33].

With these important methodological issues in mind, we will review in more detail the studies that have addressed the question of whether variation in the human genome is associated with obesity and type 2 diabetes in individuals of recent African descent in the same manner as in more extensively studied groups, and/or whether other variants might be more important. Efforts to date to find genetic causes for the differences in prevalence of these disorders have been largely disappointing; the nature of gene-environment interactions remains the focus of ongoing investigation in all groups and may be more revealing eventually. Initial approaches in African Americans have examined candidate variants or genes with the largest effects and/or biologic plausibility, including *FTO* for obesity and *TCF7L2* for T2D which has then ultimately led on to fully executed GWAS approaches in this ethnicity. 

## 3. Obesity

Excess body fat and adverse fat distribution, especially when they develop early in life, have a myriad of clinical sequelae, including diabetes and cardiovascular disease, and contribute to excess lifetime morbidity and mortality [34]. The most frequently used proxy for adiposity, body mass index (BMI, weight in kilograms divided by height in meters, squared), has the advantage of being easily, inexpensively and consistently measurable. However, the use of BMI can introduce systematic over- or underestimates of adiposity in many circumstances, including children, the elderly, athletes, and some racial/ethnic groups [35]. For example, south Asians may have consistently higher adiposity, and non-Hispanic blacks, lower adiposity, for a given BMI [36]. Differences exist even within traditionally defined racial groups; for example, immigrant girls of East African descent demonstrate higher adiposity at a given BMI than African American girls [37]. This issue also has clinical implications in estimating the interaction between BMI and mortality; similar to White women, in non-Hispanic black women, mortality was higher when BMI exceeded 25 kg/m^2^, but, in contrast, waist circumference only adversely affected mortality in women with BMI less than 25 kg/m^2^ [38]. After reviewing these and other examples, the Endocrine Society recommended the development of ethnicity-specific cut-off for central adiposity, likely framed in terms of waist circumference as opposed to BMI [30].

## 4. The “Fat Mass and Obesity Associated” (*FTO*) Locus

In individuals of European descent, a SNP in the first intron of the fat mass and obesity-associated locus (*FTO*) on chromosome 16q22.2 emerged from early investigations as very strongly associated with severe and childhood-onset obesity; in populations of European descent, the proportion of attributable risk for common obesity conferred by the high-risk haplotype has been estimated at 22% [39]. *FTO* variation appears to led to increased energy intake [40] by modifying hypothalamic control of appetite. Individuals with the minor allele of the associated SNP (rs9939609) have more fat mass, specifically in the subcutaneous adipose depot [41]. Recent work has demonstrated that not only magnitude of BMI, but also its variability is associated with variation at SNP rs7202116, further supporting a role for the *FTO* locus in mediating gene-environment interactions [42]. These two SNPs (rs9939609 and rs7202116) are correlated (*r* ^2^ is 0.967 in CEU—CEPH Utah Residents with Northern and Western European Ancestry) with individuals of European descent [12] and therefore likely do not suggest independent association.

Investigators have sought to replicate findings of an association with variation near the *FTO* locus and BMI in individuals of recent African descent, as summarized in Table 1. As noted previously, the small degree of LD in these populations makes it difficult to conclude that the absence of a detectable association stems from a true difference in biology, or potentially more likely at a locus with a consistent, replicated effect and biological plausibility, where the incorrect tagging SNP was assessed. Indeed, it has been shown that the selection of rs3751812 does result in a similar association with BMI in African American children [20] although this relationship was not detected in different individual studies [13, 22] but was replicated in a large meta-analysis [18]. Gene by ethnicity interaction was detected for rs3751812 in one study although the effect of the variation was opposite to previously published studies [16]. In the above cited studies, race/ethnicity was determined by self-report [13], or included ancestry markers of population substructure and/or principal components analysis [14–18].

More detailed mapping was performed in individuals of African American (*n* = 968) and West African (*n* = 517) (262 tag SNPs across the entire *FTO* gene) and demonstrated significant association replicated in previously tested SNPs in intron 1, and also SNPs in intron 8 and the downstream region [19]. Ancestry-informative markers (AIMs) were used to address population structure in the African Americans in this study [19]. In contrast, in a meta-analysis of six African American cohorts, no association with 10 SNPs related to *FTO* was identified [21]. Of note, the SNPs rs3751812 and rs9939609 are not typically found in association with individuals of African descent [*r* ^2^ is 0.057 in the YRI (Yoruba in Ibadan, Nigeria) population, in contrast to *r* ^2^ of 1.000 in the CEU population] [12] and therefore may reflect independent signals.

Clearly, differences in sample size, the degree of linkage disequilibrium, allele frequencies, and phenotypic heterogeneity in different racial populations contribute to the challenges in collating the diverse results of the studies referenced in Table 1. (For additional discussion of the methodological problems relevant to validation of GWAS of obesity, the reader is referred to another recent reference [43].) On the basis of its identification in the larger pediatric study, as well as adult meta-analysis, rs3751812 may be the tagging SNPs (Tables 1(b) and 2(b)) that demonstrate the degree of linkage disequilibrium between these SNP's in the YRI population). Meta-analyses of consistently measured SNPs (with phenotypes defined as closely as possible) may be additionally informative.

## 5. *FTO* and Physiology

The relationship between *FTO* genotype and T2D may also be dependent on ancestry; in whites, the risk-conferring rs9939609 A allele in *FTO* for obesity is also related to increased risk of T2D, whereas the risk-conferring rs1421085 C allele for obesity in African Americans may protect against T2D [44]; indeed, the risk association appears to be similar to whites in Asians [45]. Follow-up studies should elaborate on this intriguing result. The significance of these findings may be related to differences in modifying genes and unique population-level selection pressures; for example, opposing effects of risk-conferring variants have been understood in terms of haplotype and environmental context, including adaptation to high altitude [46]. In other studies of physiology in African Americans, risk-conferring alleles in *FTO *appear to be most related to the amount of subcutaneous adipose tissue (SAT); they may also affect glucose homeostasis [16].

## 6. Melanocortin 4 Receptor (*MC4R*)

The melanocortin 4 receptor (*MC4R*) locus is one of a number of genes that encode components of the hypothalamic leptin-melanocortin pathway, whose pleiotropic metabolic effects regulate energy balance. Mutations in *MC4R* are the most common single known cause of monogenic obesity, and variations in *MC4R* have been found in strong association with BMI in GWAS of individuals of European descent [47]. Variation in *MC4R* influences weight more than BMI, at least in part because it also appears to be associated with increased final adult height [48]. In addition to taller stature and increased appetite (hyperphagia), individuals with heterozygous or homozygous mutations in *MC4R* grow more rapidly in childhood and have higher circulating insulin levels [49]. A subset of SNPs investigated in the literature in African American individuals and individuals of recent African descent is listed in Table 2 and reviewed below.

Thirty SNPs at this locus (including rs571312, rs10871777, and rs4766282, representing perfect surrogates for the previously identified rs17782313 in individuals of European descent) were tested for their association with BMI in a key study of cohorts consisting of children of both European and African ancestry [51]. Surrogates for rs17782313 demonstrated an association with BMI in White children as White adults, but this relationship was not detected in the group of African American children. In this study, population structure was addressed using principal components analysis and ancestry-informative markers. Significant association with rs1772313 was detected in a South African population [17]. Two SNPs, rs17782313 and rs1770063, were studied in 1,890 children and adolescents; strong associations with general and visceral adiposity were found, especially in girls, but no significant interactions with racial group were present [53]. In an expanded analysis of 30 SNPs in the first pediatric cohort cited, adjusted *P* values for rs1942880 and rs12457166 were no longer significant when a more stringent statistical threshold was applied to account for multiple testing [51].

A previously identified locus in a European GWAS (SNP rs6567160) was replicated in African, African American, and Jamaican cohorts, demonstrating an effect size of 0.138 in the meta-analysis [52]. In this study, principal components analysis was used to capture population structure effects in African American populations. Two SNPs located near the *MC4R* gene were associated with BMI in one meta-analysis of six African American cohorts (rs477181 and rs4450508) [21]. Of note, the direction of the association for rs4450508 was opposite in this study compared to GWAS in European cohorts while rs477181 exhibited an association in a consistent direction. 

## 7. Candidate SNP Studies

One study referenced above included six cohorts (*n* = 4,992 subjects) of African American descent and examined nineteen loci affecting obesity identified in European cohorts. Of these, an SNP related to *MC4R* was found to be significantly associated with BMI, as reviewed above, along with SNPs related to *NEGR1*, *SH2B1/ATPA1*, and *TMEM18.* NEGR1 is highly expressed in the hypothalamus [54] and affects nerve growth [55], suggesting a role in the neurodevelopmental regulation of hypothalamic centers affecting energy balance. The *SH2B1/ATPA1* locus is also thought to be involved in the neuronal regulation of appetite and physical activity [56]. TMEM18 is known to affect neuronal migration, in addition to BMI [54, 57]. Ten SNPs in *FTO *previously associated with BMI in individuals of European descent could not be replicated in individuals of African descent, probably due to both the study's sample size and the genetic structure of the population studied [21]. In another candidate gene study, variants near *LEP* (rs10954174 and rs6966536) were found to be associated with BMI in South Africans [17]. *LEP *encodes leptin, a key adipokine that is with myriad metabolic effects, including decrease in appetite [58]. Variations in or near *LEPR*, *NPY2R*, and *POMC* were also tested, with no results that survived multiple comparison testing over the whole population [17]. Finally, in the diverse Population Architecture Using Genomics and Epidemiology (PAGE) study, including six cohorts of African American subjects (*n* = 15,415), 13 candidate loci identified in previous GWAS were screened for their association with BMI [50]. Significant associations were found for variation at *TMEM18, GNPDA2*, and *MC4R*. Additionally, two more loci related to *SHB21* and *FTO* yielded some directional evidence of association. 

In summary, candidate SNP studies have demonstrated inconsistent results in African-American populations. Where associations have been identified, these have been at loci that likely play a role in the hypothalamic regulation of appetite and metabolism. The smaller degree of linkage disequilibrium in this racial group may facilitate the identification of a more optimal tagging SNP, as illustrated for *FTO*. It is plausible that the effects of variants with known biological importance are more likely to be conserved between racial groups, and failure to replicate some of these findings may be related to study design or execution (measurement techniques and sample size) or population structure. 

## 8. GWAS/Other Approaches

A different study design used a Nigerian and African American population for gene discovery and then employed four additional African American and Jamaican cohorts for replication. This study was only sufficiently powered to detect variants of large effect (i.e., explaining > 1% of variants) in populations of recent African descent [52]. Ancestry informative markers plus imputation strategies were used in a West African population (for the Nigerian group) and also a combination of West African and Northern European (for the African American group) reference panels. Copy-number variants (CNVs) were also assessed; however, no SNPs or CNVs reached genome-wide significance for association with BMI in a total of four replication cohorts.

GWAS of BMI in African Americans showed nominally statistically significant associations (*P* < 0.0001) and replication (*P* < 0.05) at *PP13439-TMEM212, CDH12, MFAP3-GALNT10*, and *FER1L4* [59]. However, none of these reached the threshold for formal genome-wide statistical significance (*P* < 5 × 10^−8^) primarily due to small sample size. Principal components analysis was used, and ancestry-informative markers were genotyped to account for population structure. No previously identified loci in GWAS of individuals of European descent were convincingly replicated with only one SNP in *MC4R* yielding nominal significance but with an opposite direction of effect (see Table 2). 

In a genome-wide appraisal of CNVs, three copy-number variants overlapping the *PARK2*, *GYPA*, and *SGCZ* genes were associated with BMI, obesity, and obesity-related traits in the Genetic Epidemiology Network of Arteriopathy (GENOA) and Hypertension Genetic Epidemiology Network (HyperGEN) African American cohorts [60]. Finally, there is in fact some suggestion that admixture mapping may be informative in and of itself if ancestry specific markers-actually contribute to the etiology of obesity themselves [33, 61].

## 9. Type 2 Diabetes

T2D is diagnosed when any of the following criteria are met: fasting plasma glucose greater than or equal to 126 mg/dL (7.0 mmol/L), 2-hour plasma glucose greater than or equal to 200 mg/dL (11.1 mmol/L), or a random blood glucose measurement greater than or equal to 200 mg/dL (11.1 mmol/L) in the presence of classic symptoms. Recently, a hemoglobin A1C (A1C) measurement in a certified laboratory of 6.5% or more has also been considered diagnostic of T2D [62].

A key point that is relevant to the current review is that the relationship between glycemia and A1C may differ between racial groups [63]. Taking into account fasting blood glucose may improve the utility of this measurement [64], while A1C likely remains an important determinant of complications of diabetes [65], although the pitfalls of its use in isolation have been noted [66]. Interestingly, the MAGIC investigators used NHANES data to demonstrate that variants influencing A1C variation were different in frequency between racial groups, and differentially affected A1C, although not diabetes-related mortality [67]. The shortcomings of A1C as a diagnostic criterion, as well as the potential insights that can be gleaned about racial differences in this measurement, are beyond the scope of this paper, but have been addressed elsewhere [68, 69]. In the following section, we note where possible whether the diagnosis of T2D relied on A1C in the studies reviewed. 

## 10. Transcription Factor 7-Like 2 (*TCF7L2*) 

Variation within intron 3 of the transcription factor 7-like 2 (*TCF7L2*) gene [70] is very strongly associated with the development of T2D. The protein product of this gene is a transcription factor, a member of the canonical Wnt-signaling pathway whose physiologic role in glucose homeostasis may be to modulate enteroendocrine cell expression of the proglucagon gene, which encodes both glucagon and the incretin hormone, glucagon-like peptide 1 [71]. In populations of European descent the risk allele confers a population-attributable risk of type 2 diabetes of 21% [70].

Several studies led the way in investigating the role of variation in *TCF7L2* and T2D in individuals of African descent. One study in the UK examined the risk association with two intronic SNPs within *TCF7L2* (rs7903146 and rs12255372) in individuals of Indian Asian and Afro Carribean origin; in both groups, the risk allele was associated with a higher rate of T2D, but the result did not achieve statistical significance in the Afro Carribean sample [72]. Race/ancestry designation was made by a combination of self-report and answers to standardized questions in the preceding study. These same two SNPs were studied with respect to their ability to predict progression to overttype 2 diabetes in the Diabetes Prevention Program (DPP); in this study, race was designated by self-report [73] Minor allele frequencies at each of these SNPs which was similar in whites and African American's (for rs12255372, 0.32 and 0.31, and for rs7903146, 0.33 and 0.31), but lower in Hispanics (0.23, 0.24), Asians (0.14, 0.17), and American Indians (0.05, 0.12). Homozygosity for the high-risk allele at rs7903146 conferred excess risk of incident diabetes in the 497 Hispanics included, but not in the 605 African Americans or other racial groups included. Results were not significant in any non-White group for rs7903146. In one case-control study, *TCF7L2* intron 4 SNPs (rs7895340, rs11196205, and rs12255372) just reached or nearly reached statistical significance; the most significant SNPs were in *TCF7L2*: rs7903146 and rs7901695 [74]. In another case-control study (*n* = 993, *n* = 1, 054) *TCF7L2* SNP (rs7903146) was associated with T2D [75]. Ancestry-informative markers were genotyped to account for admixing in this sample.

In the multisite SEARCH for diabetes in youth study, variants in *TCF7L2* (rs12255372 and rs7903146) were genotyped in African American (*n* = 154) and non-Hispanic White (*n* = 86) children and adolescents who presented clinically with antibody-negative, monogenic DM-negative T2D between the ages of 10 and 22, as well as a population-based group of race/ethnicity-matched controls (*n* = 391 and *n* = 608, resp.) [76]. Race was self-reported, and results were adjusted for proportion of African ancestry. Neither allele frequency nor odds ratios appeared to support the hypothesis that the effect of *TCF7L2 *variation may be more pronounced in these younger subjects although its sample size may limit the study's statistical power. 

African Americans with the risk allele at rs7903146 had a higher risk of T2D in the Atherosclerosis Risk in Communities (ARIC) study; this risk appeared highest if they also had adverse metabolic profiles (obesity, low HDL cholesterol). African Americans homozygous for the risk-conferring T allele with both obesity and low HDL had an odds ratio of developing T2D of 6.04 (95% CI, 3.70–9.87), and in Caucasians, the odds ratio was 9.35 (6.72–13.00) [77]. A global meta-analysis demonstrated reproducible association between *TCF7L2* variation and individuals of North African (Morrocan) descent [78]. The authors also pooled previously published data and performed a meta-analysis demonstrating consistent odds ratios in individuals of Northern European, Asian, and African descent, for a total pooled odds ratio of 1.46 (1.42–1.51, yielding an impressive *P* of 5.4 × 10^−140^) [78].

Using tagging SNPs, the region of susceptibility to T2D was localized to a 4.3 kb interval; in individuals of African (YRI) ancestry, this region is in LD with rs7903146. To identify new SNPs that may better identify the causal variant, his area was resequenced in 96 African American individuals. Analyses suggested that rs7903146 was the trait-defining SNP in African Americans; indeed further studies now support the fact that this SNP is in fact very likely to be the causative variant at this locus [79, 80]. 

## 11. **TCF7L2 ** and Physiology

In a study that included 118 African American girls and women, homozygosity for the high-risk T allele at rs12255372 was associated with a lower disposition index (reflecting reduced compensatory modulation of the beta cell sensitivity to glucose in response to changes in peripheral insulin sensitivity); this result was observed in 138 women of European descent as well. The rs7903146 variant was not associated with metabolic traits in this study [81]. Other studies have not found associations with glucose homeostasis in African Americans [82, 83].

## 12. Candidate SNPs and Cumulative Risk of T2D

In the initial report from the PAGE study involving African Americans (1,077 cases and 1,469 controls), it demonstrated a significant association with *PPARG* (rs1801282), *IGF2BP2* (rs4402960), *JAZF1* (rs86745), as well as *TCF7L2* (rs7903146) [84]. They also note that African Americans carry more risk alleles [84]. Admixture was not included in this analysis. In the DPP, a similar cumulative risk allele score appeared associated with the development of T2D, but this result did not reach statistical significance [73]; however, admixture considerations were not included. In a follow-up study from the PAGE consortium [85], the generalizability of previously identified SNPs was investigated, and overall there was directional consistency across populations.

A case-control study tested SNPs in six known T2D genes in 577 subjects with T2D enriched for nephropathy, 596 African American control subjects, and used 70 AIMs to adjust for the potential effect of stratification [74]. ATP-sensitive inwardly rectifying potassium channel subunit Kir6.2 (*KCNJ11*) and hepatocyte nuclear factor 4 (*HNF4A*) SNPs displayed similar associations as two intronic 4SNPs in *TCF7L2*, that is, nominal or marginal significance. The authors conclude that *TCF7L2* contributes substantially to disease susceptibility in African Americans [74].

In another study, SNPs in twelve candidate loci (*TCF7L2, IDE/KIF11/HHEX, SLC30A8, CDKAL1, PKN2, IGF2BP2, FLJ39370*, and *EXT/ALX4*) were genotyped in 993 African American patients with T2D, and 1,054 control patients without T2D. Ancestry-informative markers were genotyped to account for admixture. An intragenic SNP on 11p12, rs9300039 (near *TCF7L2*), was the only variant associated with T2D after adjustment. The authors point out that four of the SNPs are monomorphic in YRI (with only the risk allele from populations of European descent present), such that their utility could be limited [75].

In a separate investigation using a case-control approach, seventeen index T2D variants were genotyped in 2,652 African American cases and 1393 controls [86]. Individual and cumulative risk allele loads were assessed. *ADAMST9* (rs4607103), *WFS1* (rs10010131), *CDKAL1* (rs10946398), *JAZF1* (rs864745), and *TCF7L2* (rs7903146) suggested an association with T2D. In these African American subjects, the previously defined risk allele in *ADAMST9* was T2D-protective; that is, the effect was opposite to that in European populations; this could reflect an independent new mutation on a different haplotype, and/or a false-positive result. Cumulative risk scoring suggested that increased allele load was related to increased T2D risk, but when *TCF7L2* genotype was included in an instrumental analysis, the cumulative allele risk score was no longer significantly predicted T2D. This is despite the fact that individually, each of the other SNPs remained associated, when accounting for either *TCF7L2* genotype or BMI. The authors posit that genetic risk may be mediated through *TCF7L2* genotype [86], and AIMs were used to perform admixture analysis and adjust for the potentially confounding effect of stratification in the previous study; admixture was found to exert a significant (*P* = 1.6 × 10^−4^) effect.

Thirty-two candidate SNPs in 1,496 African American subjects from the Genetics of NIDDM (GENNID) study [87] were assessed and demonstrated some additionally consistent results. Of the candidate SNPs tested, SNPs associated with *BCL11A* (rs10490072), *JAZF1* (rs864745), and *WFS1* (rs1001031) were significantly associated with T2D after adjusting for BMI. *BCL11A* is important for lymphoid cell development [88] and *JAZF1* is a zinc finger protein potentially important in neoplastic transformation [89]. *WFS1* encodes a transmembrane protein that is mutated in Wolfram syndrome (diabetes, optic atrophy, and deafness) [90]. Mechanistic studies of the role of candidate SNPs in glucose homeostasis have been more informative in Hispanic Americans than African Americans and demonstrate evidence mainly for defects in insulin secretion [91].

Similar to studies of obesity, investigations of the genetic variation-underlying risk of T2D in African-Americans yield results that often support the existence of conserved biological pathways (e.g., *PPARG*), potentially important effects of population-level variation or selection in exerting opposing effects (e.g., *ADAMTS2*), as well as the contribution of study design (including sample size, adjustment for admixture, or use of ancestry-informative markers) to such findings. Investigations of gene-environment interactions and/or the physiologic differences conferred by these variants will be illuminating. 

## 13. GWAS

A genome-wide association study (GWAS) conducted in African Americans that also included one replication and three validation cohorts leds to one SNP achieving genome-wide statistical significance; rs7560163 is located intergenically between *RND3* and *RBM43*. The authors point out that T2D risk was associated with the major, that is, more common allele, an unusual finding further highlighting some of the population-specific considerations important for this research [92]. A different GWAS was also been performed for traits related to glucose homeostasis and identified variants in/near *SC4MOL*, which is a gene important for lipid biosynthesis, and *TCERG1L*, a potential modulator of adiponectin, as associated with fasting insulin and insulin resistance in African Americans [93]. 

A number of studies have explored the potential source of racial differences in T2D-conferring genetic variation. In one such investigation, using ten out of twelve risk-conferring SNPs for T2D validated in five or more subpopulations, Chen et al. [94] examined their population differentiation in three separate catalogs of genetic variation. They found that risk alleles decrease in frequency from Sub-Saharan Africa through Europe to East Asia, in a way that is different from other diseases, and may explain higher genetic risk for T2D in individuals of recent African descent than of East Asian descent. They posit that this could be related to selective pressures on homeostatic mechanisms for maintaining energy balance in difference environments [94]. A frequently cited example of a disease-causing variant that may have previously exerted a survival advantage is apolipoprotein L-1 (*APOL1*); this gene encodes a factor that lyses trypanosomes and so protects from sleeping sickness, and variants that appear to confer this capacity also happen to increase the risk of kidney disease in African Americans [95]. Physiologic studies have not yet elucidated the mechanisms that might provide an analogous explanation for differential genetic variation related to diabetes risk in African Americans. For example, one study found that *TCF7L2* genotype did not explain lower insulin sensitivity and greater insulin-induced response to glucose as assessed by frequently sampled intravenous glucose tolerance test in African Americans as compared to European Americans [81].

## 14. Conclusions

In the 2012 Endocrine Society scientific statement on health disparities in endocrine disorders, the authors conclude that “There is little evidence that genetic differences contribute significantly to race/ethnic disparities in the endocrine disorders examined” [30]. Certainly, race is not biologically determined, and policy interventions that address the important social determinants of health should be prioritized [96]. However, these ideas are not inconsistent with the contention that the investigation of the genetic contributions to obesity and T2D in different racial groups is potentially informative. This paper has provided several examples where such research not only ensured fairness by promoting inclusion of groups not comprehensively studied previously, but also produced important results, for example, permitting the identification of likely common tagging SNP for *FTO* [20] and localization of the important key diabetes susceptibility variant in *TCF7L2* [97]. As may be expected, variation in genes important for conserved physiological pathways often exhibits consistent effects across populations; exceptions may illustrate unique effects of modifying genes and/or a history of population-level selection, or the need for including innovative statistical methods in follow-up work.

In the future, sufficiently powered studies and/or those leveraging new sequencing technologies have the potential to demonstrate novel associations. Moreover, racial differences do appear to exist in the association between genetic variation and T2D risk [93, 97] that justify additional investigation. Further studies, mindful of methodologic pitfalls and opportunities and attentive to ethical implications, could help to mitigate the significant global impact of obesity and T2D.

## Figures and Tables

**Table 1a:** (a)

*FTO* SNP	Population (*n*, study, reference)	Minor allele	Minor allele frequency (affected/control)	*P* value (for relationship with BMI)
rs9939609	AA children (Cincinnati) (*n = *497) [13]	A	0.48	0.87
	AA children (GCTS, LACHY, APEX) (*n = *1025) [14]	A	0.446	0.02
	AA adults (IRAS-FS) (*n = *604) [15]	A	0.491	0.014
	AA adults (IRAS) (*n = *288) [16]	A	0.418	0.71
	South African adults (birth to age 20) (*n = * 990) [17]	A	0.47	0.029/0.071
	AA adults (Jackson heart study) (*n = *4042) [18]	A	0.469	0.4125
	AA adults Maywood (*n = *775) [18]	A	0.417	0.5735
	African-derived adults (WA, AA) (*n = *1,485) [19]	A	0.504 (WA) 0.467 (AA)	0.768 (WA) 0.136 (AA)

rs8050136	AA children (CHOP) (*n = *2,002) [20]	A	0.449/0.437	
	AA children (Cincinnati) (*n = *497) [13]	A	0.43	0.87
	AA adults (IRAS-FS) (*n = *604) [15]	A	0.483	0.011
	AA adults (IRAS) (*n = *288) [16]	A	0.391	0.20
	South African adults (birth to age 20) (*n = * 990) [17]	A	0.42	0.021/0.057
	AA adults (Jackson heart study) (*n = *4,042) [18]	A	0.424	1
	AA adults (MEC) (*n = *3,482) [18]	A	0.436	0.2990
	African-derived (Jamaica Spanish Town) (*n = *1407) [18]	A	0.460	0.9899
	African-derived adults (G × E) (*n = *938) [18]	A	0.452	0.8774
	AA adults Maywood (*n = *775) [18]	A	0.472	0.6404
	Meta-analysis 6 AA cohorts (*n = *4992) [21]	A	0.45	0.141

rs3751812	AA children (CHOP) (*n = *2,002) [20]	T	0.115/0.090	0.017
	AA children (Cincinnati) (*n = *497) [13]	T	0.12	0.86
	AA adults (GenNet) (*n = *1,101) [22]	T	0.11	0.8
	AA adults (IRAS-FS) (*n = *604) [15]	T	0.143	0.38
	AA adults (IRAS) (*n = *288) [16]	T	0.083	0.22
	South African adults (birth to age 20) (*n = * 990) [17]	T		
	African-derived adults Meta-analysis (*n = *10,819) [18]	T	0.078–0.117	2.58*e* − 6

rs8057044	AA children (Cincinnati) (*n = *497) [13]	G	0.30	0.00054 (unadj)
	AA adults (IRAS-FS) (*n = *604) [15]	G	0.246	0.053
	AA adults (IRAS) (*n = *288) [16]	G	0.314	0.039
	AA adults (Jackson heart study) (*n = *4,042) [18]	G	0.283	1
	AA adults (MEC) (*n = *3,482) [18]	G	0.301	0.1476
	AA adults Maywood (*n = *775) [18]	G	0.25	0.6121
	Meta-analysis 6 AA cohorts (*n = *4,992) [21]	G	0.27	0.710
	African-derived adults (WA, AA) (*n = *1,485) [19]	G	0.27 (WA) 0.27 (AA)	NS

rs1108102	AA adults (IRAS) (*n = *288) [16]	A	0.125	5.4*e* − 4
	Meta-analysis 6 AA cohorts (*n = *4,992) [21]	A	0.15	0.636
	African-derived adults (WA, AA) (*n = *1,485) [19]	A	0.186	0.142

rs17817449	South African adults (birth to age 20) (*n = * 990) [17]	G	0.37	0.008/0.022
	AA adults Maywood (*n = *775) [18]	G	0.361	0.1513
	African-derived adults (WA, AA) (*n = *1,485) [19]	G	0.409 (WA) 0.396 (AA)	0.554 (WA) 0.151 (AA)

rs9941349	African-derived adults meta-analysis (*n = *10,819) [18]	T	0.167–0.191	3.61*e* − 6
	African-derived adults (WA, AA) (*n = *1,485) [19]	T	0.165 (WA) 0.187 (AA)	NS

**Table 1b:** (b)

*r* ^2^ (YRI)	rs9939609	rs8050136	rs3751812	rs8057044	rs1108102	rs17817449	rs9941349
rs9939609	1						
rs8050136	0.843	1					
rs3751812	0.057	0.068	1				
rs8057044	0.065	0.119	0.014	1			
rs1108102	0.030	0.050	0.016	0.044	1		
rs17817449	0.621	0.736	0.092	0.151	0.024	1	
rs9941349	0.009	0.033	0.356	0.000	0.018	0.005	1

**Table 2a:** (a)

*MC4R* SNP	Population (*n*, study, reference)	Minor allele	Minor allele frequency	*P* value for association with BMI
rs17782313	Meta-analysis, 6 AA cohorts (*n = *4,992) [21]	C	0.29	0.082
	South African adults (birth to age 20) (*n = *990) [17]	C	0.25	0.045
	African decent, adults (PAGE) [50]	C	0.29	0.04
	Meta-analysis, 6 AA cohorts (*n = *4,992) [21]			0.02 (Effect opposite risk allele)
rs571312	AA children CHOP (*n = *3,723) [51]	T	0.335	0.348
rs10871777	AA children CHOP (*n = *3,723) [51]	G	0.286	0.161
rs476828	AA children CHOP (*n = *3,723) [51]	G	0.412	0.825
rs1942880	AA children CHOP (*n = *3,723) [51]	C	0.499	0.008
rs12457166	AA children CHOP (*n = *3,723) [51]	A	0.230	0.013
rs633265	Meta-analysis 6 AA cohorts (*n = *4,992) [21]	C	0.22	0.455
	AA children CHOP (*n = *3,723) [51]	C	0.248	0.075
rs477181	Meta-analysis 6 AA cohorts (*n = *4992) [21]	G	0.47	0.018
	AA children CHOP (*n = *3,723) [51]	G	0.492	0.252
rs4450508	Meta-analysis, 6 AA cohorts (*n = *4,992) [21]	A	0.30	0.046
rs6567160	AA (*n = *743), Nigerian (*n = *1,188) [52]	C (reference allele)		0.0035
	AA adults (GCI) (*n = *494) [52]	C (reference allele)		0.227
	AA adults (Maywood) (*n = *756) [52]	C (reference allele)		0.024
	African-derived adults (Jamaica G × E) (*n = *959) [52]	C (reference allele)		0.497
	African-derived adults (Jamaica SPT) (*n = *1,478) [52]	C (reference allele)		0.021

**Table 2b:** (b)

*r* ^2^ (YRI)	rs17782313	rs571312	rs10871777	rs476828	rs1942880	rs12457166	rs633265	rs477181	rs4450508	rs6561760
rs17782313	1									
rs571312	0.258	1								
rs10871777	0.924	0.204	1							
rs476828	0.503	0.472	0.544	1						
rs1942880	0.206	0.121	0.227	0.241	1					
rs12457166	0.000	0.001	0.001	0.004	0.074	1				
rs633265	0.064	0.083	0.069	0.127	0.150	0.041	1			
rs477181	0.140	0.118	0.166	0.329	0.013	0.255	0.161	1		
rs4450508	0.131	0.002	0.184	0.340	0.008	0.103	0.067	0.413	1	
rs6561760	0.086	0.012	0.068	0.000	0.172	0.068	0.035	0.007	0.042	1
